# Evaluation of functional arterial spin labeling data using a perfusion template

**DOI:** 10.1186/1471-2202-12-S1-P9

**Published:** 2011-07-18

**Authors:** Jan Petr, Elise Bannier, Hélène Raoult, Jean-Christophe Ferré, Jean-Yves Gauvrit, Christian Barillot

**Affiliations:** 1 Neuroradiology Dept., University Hospital of Rennes, F-35043 Rennes, France; 2 INRIA, VisAGeS Project-Team, F-35042 Rennes, France; 3 INSERM, U746, F-35042 Rennes, France; 4 University of Rennes I, CNRS, UMR 6074, IRISA, F-35042 Rennes, France; 5 Neurinfo Platform, University Hospital of Rennes, F-35043 Rennes, France

## 

ASL allows non-invasive imaging and quantification of brain perfusion by magnetically labeling blood in the brain-feeding arteries. In this study, a template created from perfusion images of 25 resting healthy subjects was used to automatically detect hyper perfusion patterns of 8 other subjects. DARTEL registration was used to improve the precision of the template and partial volume correction to prevent interpolation artifacts.

MR imaging was performed on a 3T MR scanner with a 32-channel head coil and consisted in a 1x1x1mm^3^ 3D T1 and a PICORE Q2TIPS ASL with 3x3x7mm^3^ pixel size, TR/TE=3000/25ms, TI=1700ms and Q2TIPS saturation [3] at 700ms. ASL images were acquired in 25 healthy subjects (mean age 31.6). Additionally, 8 healthy right-handed subjects underwent fASL study following a bloc-design experiment with seven interleaved 30s-phases of rest and motor task of the dominant hand. The following processing was applied: a) Perfusion was quantified [2]; b) ASL images were co-registered with their T1 images; c) T1 images were segmented to GM/WM regions; d) Partial volume effects in ASL images were corrected for using high-resolution segmentation [4]; e) T1 images were aligned to the ICBM-152 template [5]; f) T1 images were aligned using DARTEL registration [1]; g) ASL images were spatially normalized using the transformations from e,f); h) The perfusion template was created as the mean and variance of the spatially normalized ASL images of the 25 resting subjects. For each of the 8 fASL subjects, the activated image was created by averaging the images acquired during activity phases. Hyperperfused areas were identified by comparison with the template (p<0.001). To examine false positive hyperperfusion detection, hyper-perfusion areas were assessed on each of the 25 patients using a template created from the 24 remaining patients. Standard fASL processing was performed by using SPM8 using 6-mm FWHM Gaussian spatial smoothing and GLM model with statistical significance p<0.001. Regions corresponding to primary motor and supplementary motor cortex were manually selected and were used to evaluate the results of template comparison. The ratio of hyperperfusion false positive detection was below 1% for all subjects. The hyperperfused areas obtained with template comparison represented 86,0±10,1% for the primary motor cortex region and 79,9±9,3% for the supplementary motor cortex of the ground-truth obtained by standard Bayesian analysis. Using the Bayesian model, the supplementary motor cortex was not detected at all in one patient and this dataset was not taken into account. Figure 1.

**Figure 1:**
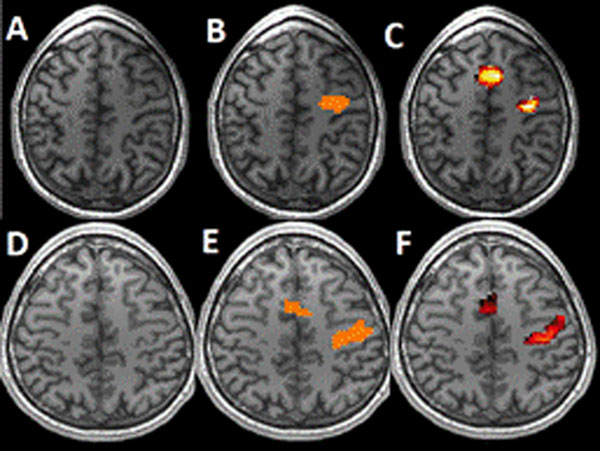
Task-related activation zones of two patients. **A, D**. T1 image. **B, E**. SPM detection. **C, F**. Comparison with template.

## Conclusions

This study shows that a perfusion template can be used to assess task-related activation zones in functional ASL data while using only activated phase. Two assumptions can be made to explain why standard functional analysis yields slightly larger activation regions. First, the use of FWHM 6mm Gaussian kernel possibly enlarges the detected zones. Second, the data analyzed using SPM contains both resting and activated phases whereas only the activated phase was compared to the template. Future work will focus on detection of hyperperfusion in different neurodegenerative diseases taking into account registration issues of pathological T1 images.
